# A prediction model of clinical outcomes in prolonged disorders of consciousness: A prospective cohort study

**DOI:** 10.3389/fnins.2022.1076259

**Published:** 2023-02-03

**Authors:** Qi Xiong, Kai Le, Yong Wang, Yunliang Tang, Xiaoyang Dong, Yuan Zhong, Yao Zhou, Zhen Feng

**Affiliations:** ^1^Department of Rehabilitation Medicine, The First Affiliated Hospital of Nanchang University, Nanchang, Jiangxi, China; ^2^Department of Medical Oncology, The First Affiliated Hospital of Nanchang University, Nanchang, Jiangxi, China

**Keywords:** disorder of consciousness, brain injury, minimally conscious state, vegetative state, cohort

## Abstract

**Objective:**

This study aimed to establish and validate a prediction model for clinical outcomes in patients with prolonged disorders of consciousness (pDOC).

**Methods:**

A total of 170 patients with pDOC enrolled in our rehabilitation unit were included and divided into training (*n* = 119) and validation sets (*n* = 51). Independent predictors for improved clinical outcomes were identified by univariate and multivariate logistic regression analyses, and a nomogram model was established. The nomogram performance was quantified using receiver operating curve (ROC) and calibration curves in the training and validated sets. A decision curve analysis (DCA) was performed to evaluate the clinical usefulness of this nomogram model.

**Results:**

Univariate and multivariate logistic regression analyses indicated that age, diagnosis at entry, serum albumin (g/L), and pupillary reflex were the independent prognostic factors that were used to construct the nomogram. The area under the curve in the training and validation sets was 0.845 and 0.801, respectively. This nomogram model showed good calibration with good consistency between the actual and predicted probabilities of improved outcomes. The DCA demonstrated a higher net benefit in clinical decision-making compared to treating all or none.

**Conclusion:**

Several feasible, cost-effective prognostic variables that are widely available in hospitals can provide an efficient and accurate prediction model for improved clinical outcomes and support clinicians to offer suitable clinical care and decision-making to patients with pDOC and their family members.

## 1. Introduction

Disorders of consciousness (DOC) is a state in which an individual's consciousness has been severely affected due to massive brain injury. There are two essential elements of consciousness: arousal and content. Disruption of one or both of these elements may result in DOC. Prolonged disorder of consciousness (pDOC) is defined as a coma condition usually lasting more than 4 weeks (Giacino et al., 2018b). Approximately 10–15% of individuals (Andriessen et al., 2011) develop pDOC after acquiring brain injuries or experiencing nervous system dysfunction and remain in the vegetative state/unresponsive wakefulness syndrome (VS/UWS—patients open their eyes but show no clinical evidence of consciousness; Laureys et al., 2010) or in a minimally conscious state (MCS—patients showing minimal, inconsistent but clearly discernible intentional and non-reflexive behaviors such as fixation, visual pursuit, localization to noxious stimuli, reproducible movements to command, and automatic motor responses; Giacino et al., 2002). In the United States, ~100,000–300,000 patients are diagnosed with pDOC (Giacino et al., 2018a), whereas in Europe the prevalence ranges from 0.2 to 6.1 patients per 100,000 (van Erp et al., 2014). There are at least 300,000 to 500,000 patients with disorders of consciousness in China, with more than 70,000 new patients diagnosed every year and an annual cumulative medical expenditure of RMB ¥30 to 50 billion (Chen et al., 2020). Within a year after disorders of consciousness, 35% of the affected population faced mortality and only 40% had an improved consciousness (Nekrasova et al., 2021). With high morbidity and mortality, heavy economic burden, and uncertain clinical effects, disorders of consciousness bring many social and economic problems to clinical decision-making and heavy economic and spiritual burden to patients' families.

To the best of our knowledge, there are a few prognostic prediction models for patients with pDOC (Kang et al., 2022). Many previous studies aimed at identifying the risk factors affecting the prognosis of pDOC. Previous studies using functional connectivity analysis showed that the number and strength of cortical functional connections between EEG segments (Fingelkurts et al., 2013) and high frontoparietal theta and alpha coherence (Schorr et al., 2016) were associated with favorable outcomes in DOC patients. Bai et al. (2019) demonstrated that lower frontal quadratic phase self-coupling at the theta band indicated a higher probability of consciousness recovery. A study on sleep EEG indicated that the occurrence of sleep spindles was related to clinical improvement within 6 months (Cologan et al., 2013). The predictive value of the presence of mismatch negative (MMN; Kotchoubey et al., 2005), N400 (Steppacher et al., 2013), and P300 latency (Cavinato et al., 2011) on event-related potentials were found in patients with DOC. Moreover, low thalamocortical connectivity during functional magnetic resonance imaging (fMRI) (Chen et al., 2018) and low gray matter/white matter (GM/WM) ratio (Scarpino et al., 2018) are associated with poor neurological deficit in patients with pDOC. Although a number of studies identified several neurophysiologic or neuroimaging risk factors for patients with DOC, the above examinations are difficult to perform, relatively expensive, and time-consuming, which limits their wide applications.

Several previous studies showed that age, MCS diagnosis (Estraneo et al., 2019), and the presence of pupil reflex (Lee et al., 2010) were associated with better clinical outcomes. Young individuals have better neuroplasticity after brain injury, which may affect the development of DOC. In addition, patients diagnosed with MCS and pupil reflex have relatively mild brain damage after a traumatic or non-traumatic brain injury compared to those diagnosed with VS and absence of pupil reflex. Hypoalbuminemia is common in patients with DOC after severe brain injury (Morotti et al., 2017). Albumin, synthesized in the liver, plays important roles in maintaining normal metabolism functions, such as detoxification and maintaining plasma colloid osmotic pressure, which could avoid much accumulation of fluid in tissue spaces and can act as a carrier of drugs and hormones (Montalcini et al., 2015). Albumin is an indicator of protein nutrition status, and a low concentration of serum albumin is a sign of unstable clinical states (Montalcini et al., 2015). Serum albumin has been found to be a good predictor of the prognostication of cancer (Wang et al., 2022) and cognitive decline in Parkinson's disease (Shen et al., 2022). A cohort study of multiple sclerosis found that cerebrospinal fluid/serum albumin is an independent variable for the prognostication of multiple sclerosis (Berek et al., 2022). Low levels of serum albumin could aggravate the degree of brain edema and affect drug efficacy in patients after traumatic brain injury (Jungner et al., 2010). Albumin has been found to be associated with clinical outcomes in patients with traumatic brain injury (Wang et al., 2020) and ischemic stroke (Lu et al., 2022), but its predictive value of short-term clinical outcomes in patients with pDOC remains uncertain.

Thus, this prospective cohort study aimed to investigate a prognostic model based on clinical factors (i.e., clinical indices, repeated diagnosis, and serologic markers).

## 2. Methods

### 2.1. Participants and inclusion and exclusion criteria

For this prospective study, we screened patients with DOC who were consecutively admitted to a neurorehabilitation unit from January 2021 to June 2022. The inclusion criteria were as follows: (1) clinical diagnosis of VS/UWS or MCS according to the standard criteria (Giacino et al., 2004; Wannez et al., 2017); (2) age ≥ 18 years; (3) traumatic, vascular, or anoxic etiology; and (4) duration of DOC ranging from 1 to 3 months (Estraneo et al., 2020). The exclusion criteria were as follows: (1) nervous system dysfunction or unstable clinical condition (e.g., severe heart or respiratory failure); (2) previous history of brain injury or neurodegenerative diseases; and (3) patients with motor impairment and locked-in syndrome. Enrollment of the patients was carried out by three doctors involved in the study who were also responsible for patient management. During their hospital stay, comprehensive multidisciplinary rehabilitation care was offered to all patients. We divided the patients into two independent sets: 119 patients treated between January 2021 and December 2021 constituted the training set, whereas 51 patients treated between January 2022 and June 2022 constituted the validation set.

### 2.2. Sample size

We relied on an Events Per Variable criterion (EPV), notably EPV≥10, to determine the minimal sample size required (van Smeden et al., 2019). In our study, with four independent variables selected and 40% of patients with clinical improvement (Nekrasova et al., 2021), the minimal sample size calculated was 100. Allowing for a 20% dropout rate during the study, a minimum total of 120 participants were needed in the training set.

### 2.3. Data collection and clinical assessment

Upon study entry, the prehospital information about patients' clinical indices (CI), including demographic data (e.g., age, education, and sex) and medical history (e.g., pupillary light reflex, etiology, duration of DOC, hypertension, and diabetes), were collected. Pupillary light reflex was classified into two categories based on the presence or absence of bilateral light reflex. After study entry, the diagnoses were confirmed by repeated Coma Remission Scale-revised assessment (CRS-R) (at least five times a week), which is standardized by the clinical criteria for DOC. We also collected the serum albumin and hemoglobin concentrations of the patients upon study entry. Serum albumin and hemoglobin were measured from venous blood within the first 24 h of admission. The patients were followed up at 6 months after brain injury using repeated CRS-R evaluations that were performed by the same clinicians during their hospital stay. At the time of assessing the level of consciousness of patients with pDOC, drugs such as sedation, antiepileptics, and neuroexcitation were excluded.

### 2.4. Outcome definition

The primary outcome was defined by an improvement in clinical diagnosis, which was determined by repeated CRS-R assessment, the golden standard for the diagnosis of pDOC (Giacino et al., 2004; Wannez et al., 2017). While VS/UWS is a condition in which patients open their eyes but show no clinical evidence of consciousness (Laureys et al., 2010), the most frequent signs of consciousness in MCS patients are fixation, visual pursuit, localization to noxious stimuli, reproducible movements to command, and automatic motor responses (Giacino et al., 2002). Emergence from MCS is defined as the patient communicating with others (i.e., the family members or the doctors) exactly or the ability to use objects (Machado et al., 2010). The VS/UWS state is the lowest level of consciousness, while emergence from MCS is the highest level of consciousness in patients with DOC. We classified the primary outcome as improved if there was an improvement in clinical diagnosis upon follow-up compared to that upon diagnosis after study entry (e.g., patient in VS/UWS at study entry who improved to MCS or emergence from MCS and patient in MCS at study entry who emerged from MCS). We classified the clinical outcome as not improved if the clinical diagnosis did not improve (i.e., patient in VS/UWS at study entry who persisted or died and patient in MCS at study entry who worsened to VS/UWS or died or remains in MCS).

### 2.5. Statistical analysis

Continuous variables were expressed as mean ± standard deviation, and categorical variables were expressed as counts and/or frequencies. Continuous variables were subjected to the Shapiro–Wilk test for normality tests. As scores of several variables significantly varied from the norm, we compared the basic clinical characteristics between the training and validation sets by the nonparametric Mann–Whitney *U-*test for continuous variables (i.e., CRS-R and GCS total scores) and the Chi-square test for categorical variables. A univariate logistic regression analysis was performed to screen for statistically significant variables that were associated with the clinical outcomes. A multivariate logistic regression analysis was used to further analyze all statistically significant indicators in the univariate analysis. The independent prognostic variables were determined as significant if the *p* value was <0.05 in the multivariate analysis. We integrated these four independent variables into the nomogram model. The nomogram performance was quantified using the area under the curve (AUC) of the receiver operating curve (ROC) and the calibration curves in the training and validated sets. A decision curve analysis (DCA) was performed to evaluate the clinical usefulness of this nomogram model. The value of (true positive + true negative)/total was evaluated for accuracy. All the analyses were performed using the statistical package R (http://www.R-project.org, The R Foundation) and Empower (R) (www.empowerstats.com; X&Y Solutions, Inc). A two-sided *p-*value of <0.05 was considered to be statistically significant.

### 2.6. Protocol approvals

The study was performed with the approval of the First Affiliated Hospital of Nanchang University Ethics Committee (No. 2020-061-3). Written informed consent was obtained from the relatives or legal guardians of all patients.

## 3. Results

### 3.1. Description of the sample

**Figure 1:**
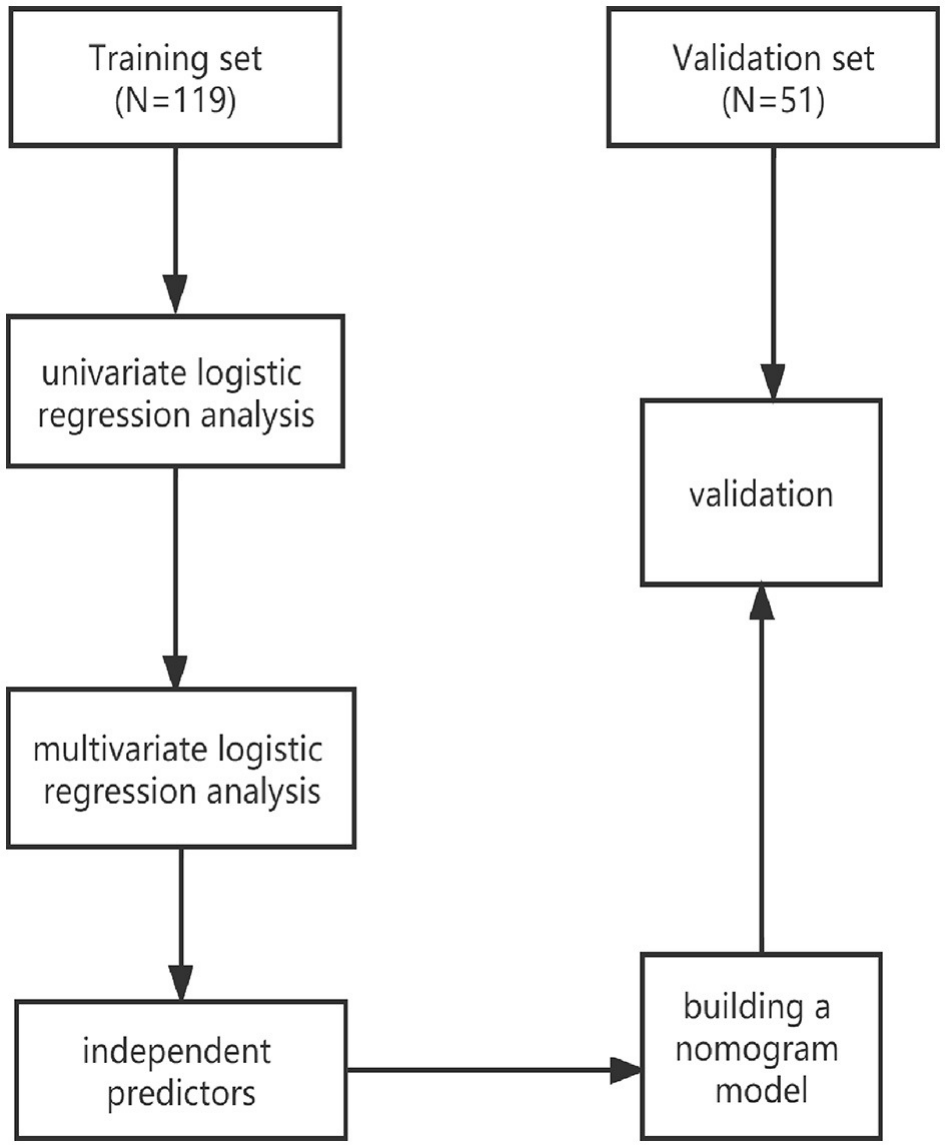
Study flowchart.

The study flowchart is presented in Figure 1. The demographic and clinical basic characteristics of the training and the validation cohorts are presented (Table 1). Overall, 170 patients (mean age 54.32 ± 14.80 years, VS 90, and MCS 80) were included. In the development set, we included 119 consecutive patients with pDOC, of whom 60 (50.42%, mean CRS-R 7.3 ± 1.93) patients showed improved clinical outcomes and 59 patients (49.58%, mean CRS-R 5.73 ± 1.74) showed poor clinical outcomes. While in the validation set, 51 patients were screened, with 29 (56.86%, mean CRS-R 8.41 ± 3.78) patients showing improved outcomes and 22 patients (43.14%, 5.77 ± 1.73) presenting poor clinical outcomes compared to baseline characteristics. There were no significant differences between the two cohorts in terms of CRS-R, GCS, age, etiology, DOC duration, sex, marital status, serum albumin level, hemoglobin level, hypertension, and diabetes.

**Table 1:** Demographic and clinical characteristics of patients with pDOC.

	**Total**	**Validation set**	**Training set**	***P*-value**
**N**	170	119	51	
Age (y)	54.32 ± 14.80	53.09 ± 15.23	57.18 ± 13.44	0.099
Serum albumin (g/L)	35.34 ± 5.63	35.65 ± 6.00	34.64 ± 4.61	0.285
Hemoglobin (g/L)	100.92 ± 15.70	102.18 ± 15.16	97.98 ± 16.66	0.110
GCS	7.81 ± 1.95	7.68 ± 1.98	8.10 ± 1.87	0.202
CRS-R	6.75 ± 2.48	6.52 ± 2.00	7.27 ± 3.32	0.070
DOC duration (d)	49.86 ± 31.88	49.24 ± 22.42	51.31 ±35.38	0.698
**Diagnosis**				0.180
MCS	80(47.05%)	60 (50.42%)	20 (39.22%)	
VS/UWS	90(52.95%)	59 (49.58%)	31 (60.78%)	
**Improved outcome**				0.441
Yes	81 (47.65%)	59 (49.58%)	22 (43.14%)	
No	89 (52.35%)	60 (50.42%)	29 (56.86%)	
**Pupillary reflex**				0.131
Absent	59 (34.71%)	37 (31.09%)	22 (43.14%)	
Present	111 (65.29%)	82 (68.91%)	29 (56.86%)	
**Sex**				0.404
Female	62 (36.47%)	41 (34.45%)	21 (41.18%)	
Male	108 (63.53%)	78 (65.55%)	30 (58.82%)	
**Job**				0.041
No	70 (41.18%)	55 (46.22%)	15 (29.41%)	
Yes	100 (58.82%)	64 (53.78%)	36 (70.59%)	
**Married**				0.109
No	16 (9.41%)	14 (11.76%)	2 (3.92%)	
Yes	154 (90.59%)	105 (88.24%)	49 (96.08%)	
**Education**				0.001
Primary	91 (53.53%)	53 (44.54%)	38 (74.51%)	
Middle	58 (34.12%)	46 (38.66%)	12 (23.53%)	
High	21 (12.35%)	20 (16.81%)	1 (1.96%)	
**Etiology**				0.198
Traumatic	73 (42.94%)	49 (41.18%)	24 (47.06%)	
Vascular	75 (44.12%)	51 (42.86%)	24 (47.06%)	
Anoxic	22 (12.94%)	19 (15.97%)	3 (5.88%)	
**Diabetes**				0.961
No	147 (86.47%)	103 (86.55%)	44 (86.27%)	
Yes	23 (13.53%)	16 (13.45%)	7 (13.73%)	
**Hypertension**				0.263
No	99 (58.24%)	66 (55.46%)	33 (64.71%)	
Yes	71 (41.76%)	53 (44.54%)	18 (35.29%)	

### 3.2. Screening prognostic predictors

In the training cohort, univariate analysis showed that improved clinical outcomes were associated with the total scores of CRS-R, GCS, age, the concentration of serum albumin, entry diagnosis, and pupillary reflex. Multivariate logistic regression analysis of improved clinical outcomes indicated that age, pupillary reflex, entry diagnosis, and the concentration of serum albumin (g/L) were the prognostic predictors for patients with pDOC. A final logistic regression analysis including the four predictors was conducted to demonstrate the odds ratio (OR) of each predictor in the model (Table 2).

**Table 2:** Univariate and multivariate logistic regression analyses.

**Variable**	**Univariable**			**Multivariable**	
	**Ref**	**OR (95%CI)**	***P*-value**	**OR (95%CI)**	***P*-value**
Age (y)	–	0.96 (0.94, 0.99)	0.002	0.94 (0.90, 0.98)	0.005
DOC duration (d)	–	1.01 (0.99, 1.02)	0.48		
CRS-R	–	1.61 (1.27, 2.04)	0.001	1.41 (0.90, 2.22)	0.134
GCS	–	1.47 (1.19, 1.83)	0.000	1.02 (0.61, 1.70)	0.936
Sex (f/m)	Female	0.95 (0.45, 2.03)	0.899		
Job (y/n)	No	1.91 (0.92, 3.96)	0.083		
Married (y/n)	No	1.41 (0.46, 4.35)	0.548		
**Education**					
Primary	Primary	1.0			
Middle		1.7 (0.8, 3.8)	0.184		
High		1.0 (0.4, 2.8)	0.983		
**Etiology**					
Traumatic	Traumatic	1.0			
Vascular		1.27 (0.58, 2.79)	0.548		
Anoxic		1.26 (0.43, 3.63)	0.673		
Diagnosis (VS/MCS)	MCS	0.09 (0.04, 0.22)	0.001	0.12 (0.04, 0.42)	0.000
Serum albumin (g/L)	–	1.09 (1.02, 1.17)	0.012	1.13 (1.03, 1.25)	0.012
Hemoglobin (g/L)	–	1.02 (0.99, 1.04)	0.146		
Pupillary reflex	Absent	4.22 (1.80, 9.88)	0.000	3.70 (1.00, 13.67)	0.049
Hypertension (y/n)	No	1.19 (0.58, 2.45)	0.637		
Diabetes (y/n)	No	0.98 (0.34, 2.81)	0.971		

### 3.3. Development and validation of the model

**Figure 2:**
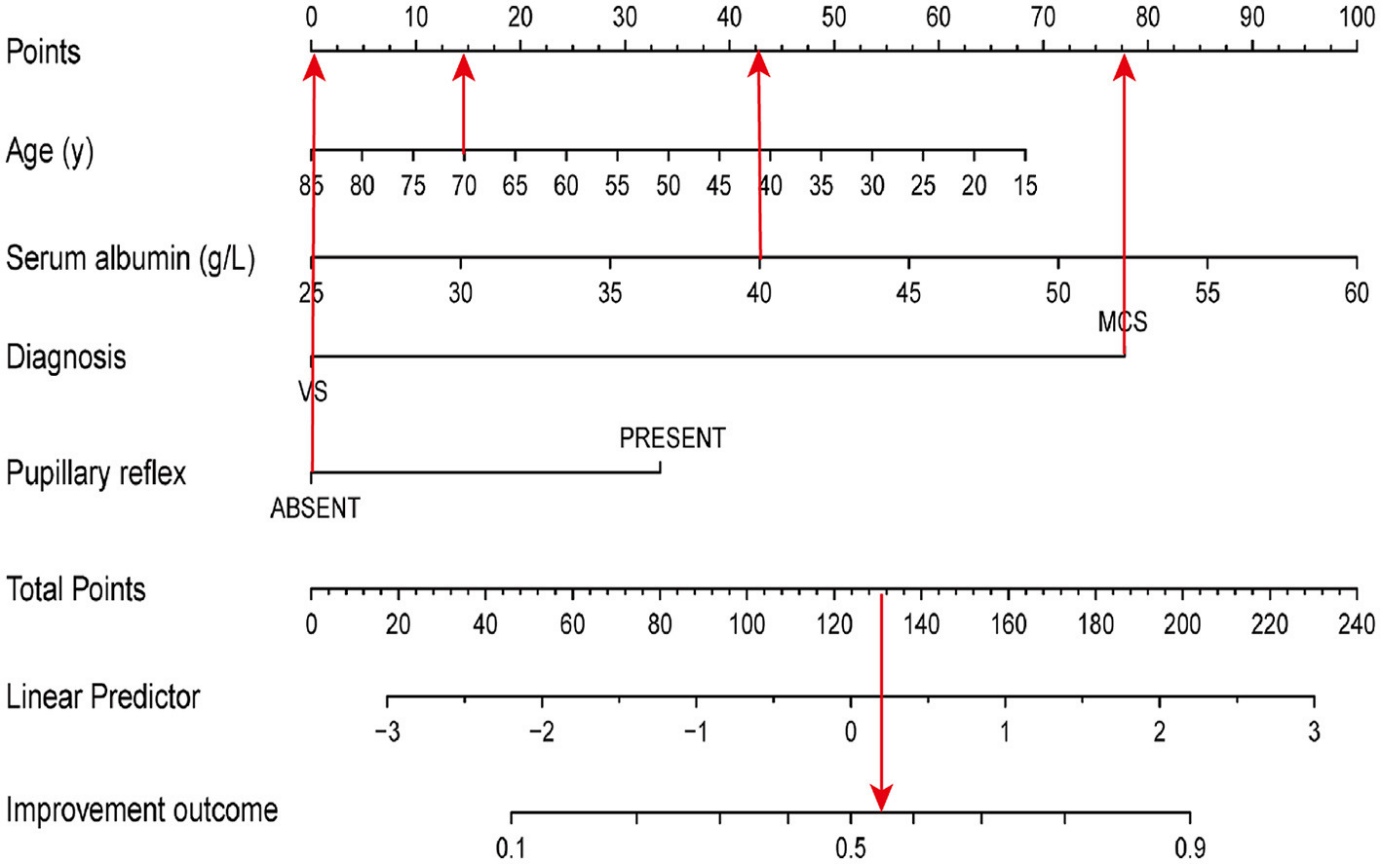
A nomogram model to predict the clinical outcomes. The red arrows present the calculation of total points of a 70-year-old patient diagnosed with MCS, absence of bilateral light reflex, and a serum albumin concentration of 40 g/L upon entry within 2 months of injury.

According to the results of the multivariate logistic regression analysis, four predictive variables (age, entry diagnosis, serum albumin level (g/L), and pupillary reflex) were used to establish a 6-month outcome prediction nomogram model in patients with pDOC (Figure 2). The total risk points were calculated by summing the points of each predictor. The higher the total points, the higher the likelihood of an improved clinical outcomes. For example, consider a 70-year-old patient diagnosed with MCS showing the absence of bilateral light reflex and a serum albumin concentration of 40 g/L upon entry within 2 months after injury. The points for age, MCS diagnosis, absence of pupil reflex, and level of serum albumin level of 40 mg/L were 15, 77.5, 0, and 42.5, respectively. The total points added up to 135, which demonstrated about a 58% likelihood of an improved diagnosis 6 months after injury.

The validation was performed by using the other cohort of 51 patients with pDOC. In the validation cohort, the independent risk factors integrated into the nomogram were examined. The accuracy of the model is 0.745 [(true positive + true negative)/total]. The AUC values were 0.845 and 0.801 in the training and validation sets, respectively.

### 3.4. Calibration and decision curve

**Figure 3:**
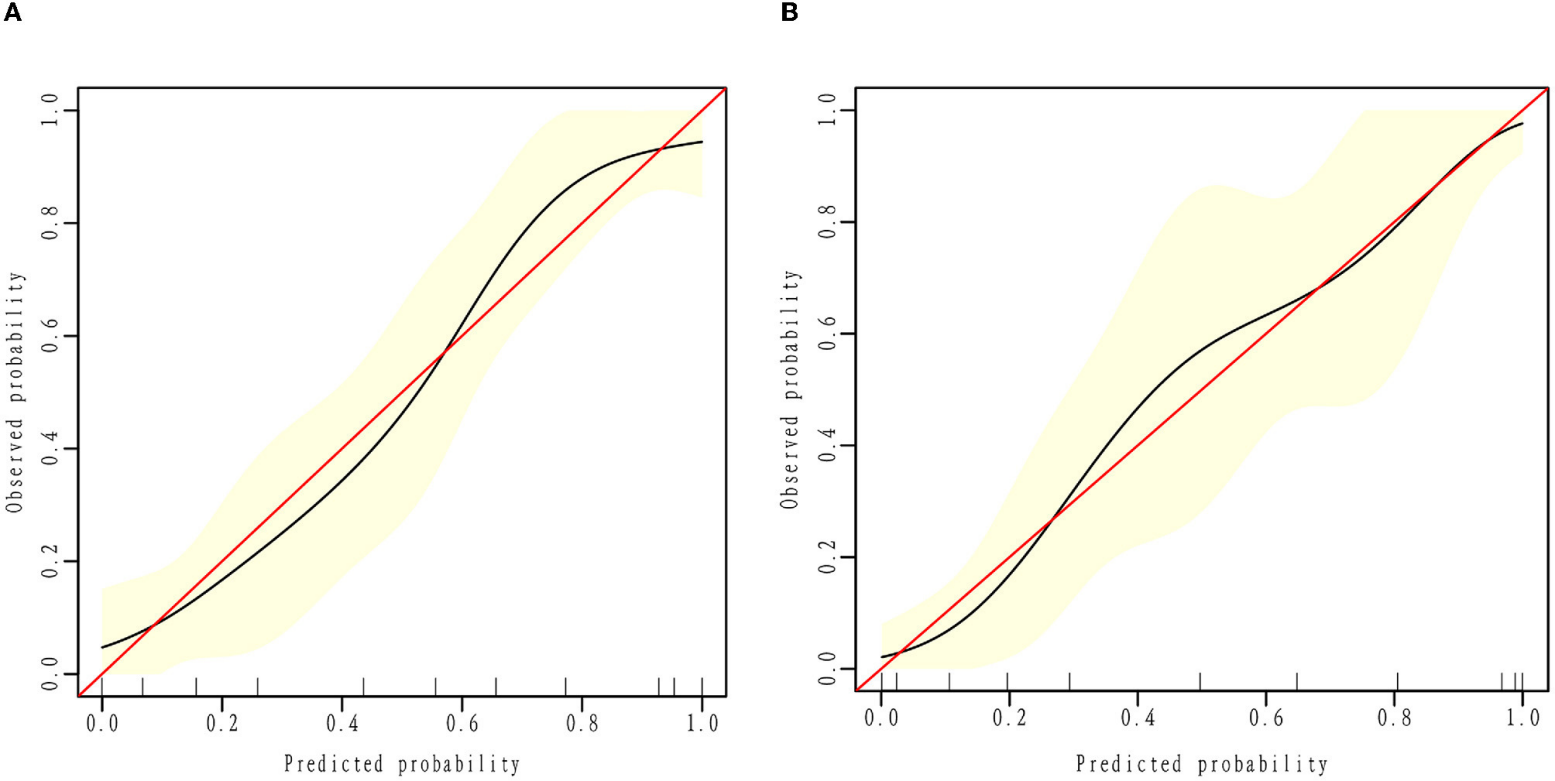
Calibration curve. The black calibration curve shows the predicted proportion of clinical outcomes against the actual probability of outcomes. The red line demonstrates the actual risk equal to the predicted risk. Calibration plots for estimating improved outcome probabilities are presented for the training **(A)** and validation cohorts **(B)**.

**Figure 4:**
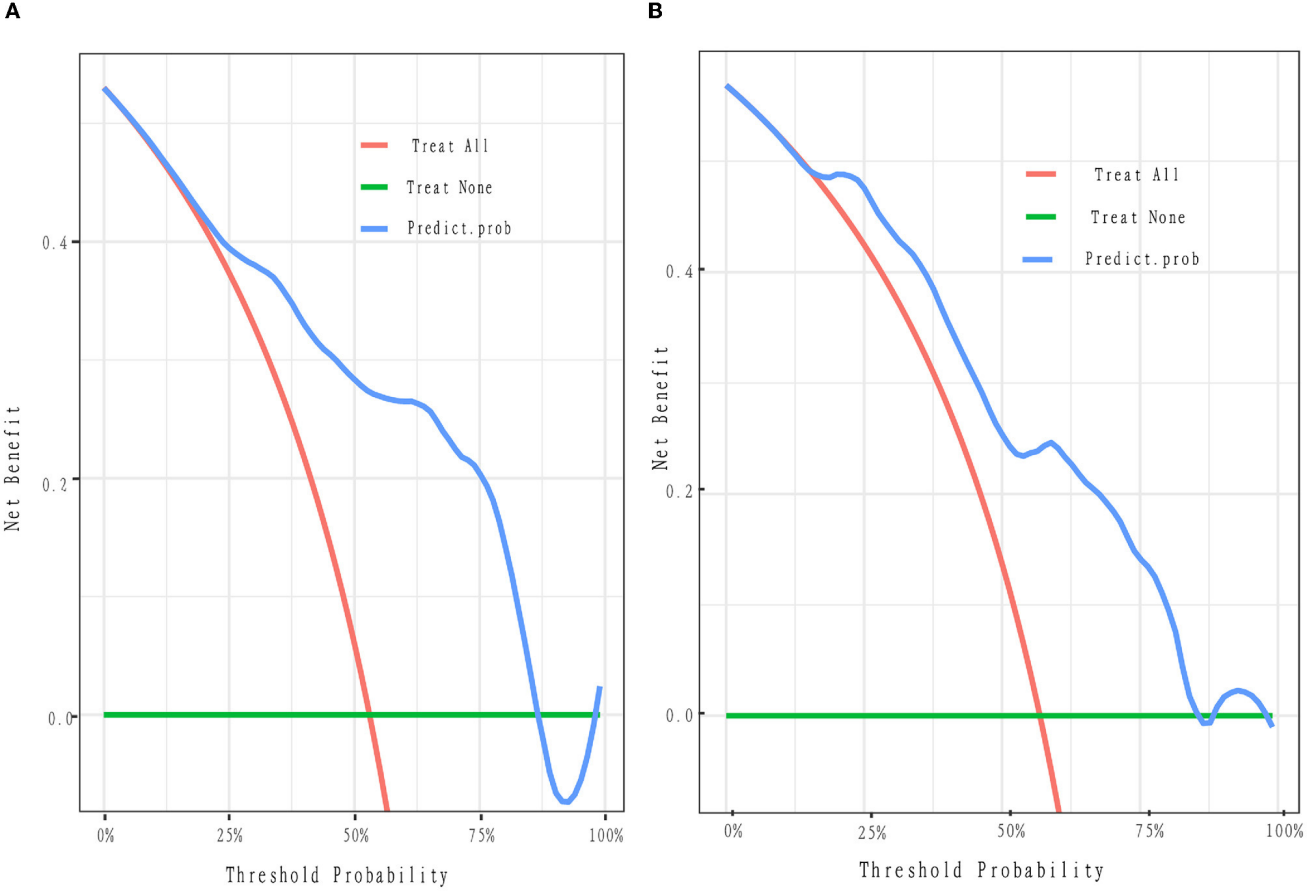
Decision curve. The y-axis represents the net benefit. The x-axis is the threshold probability. The blue curve represents the nomogram. The red oblique represents the hypothesis that all patients with pDOC have improved outcomes with all treatments. The green line represents the hypothesis that no patients will have improved outcomes. The threshold probability is where the expected benefit of the treatment is equal to the expected benefit of no treatment. For example, if the possibility of the improved outcome of a patient is over the threshold probability, then the patients with pDOC will not benefit from all the treatment strategy. **(A, B)** Depict the decision curves of the training and validation cohorts, respectively. Net benefit means the clinical efficacy of the treatment.

To evaluate the consistency of the actual and predicted risks by the model, a calibration curve was drawn to measure calibration, which is an important aspect of the prediction models. It is demonstrated that the actual risk (red line) was consistent with the predicted risk (black curve) in both the training and validation cohorts (Figures 3A, B). From the decision curve, it can be seen that the net benefit of the model (blue curve) was higher than that with all treatment at the same threshold probability (red curve) in both the training and validation sets (Figures 4A, B), which indicates that clinicians could benefit from this model for decision-making.

### 3.5. Comparison of age, pupil reflex, and diagnosis with our model

**Figure 5:**
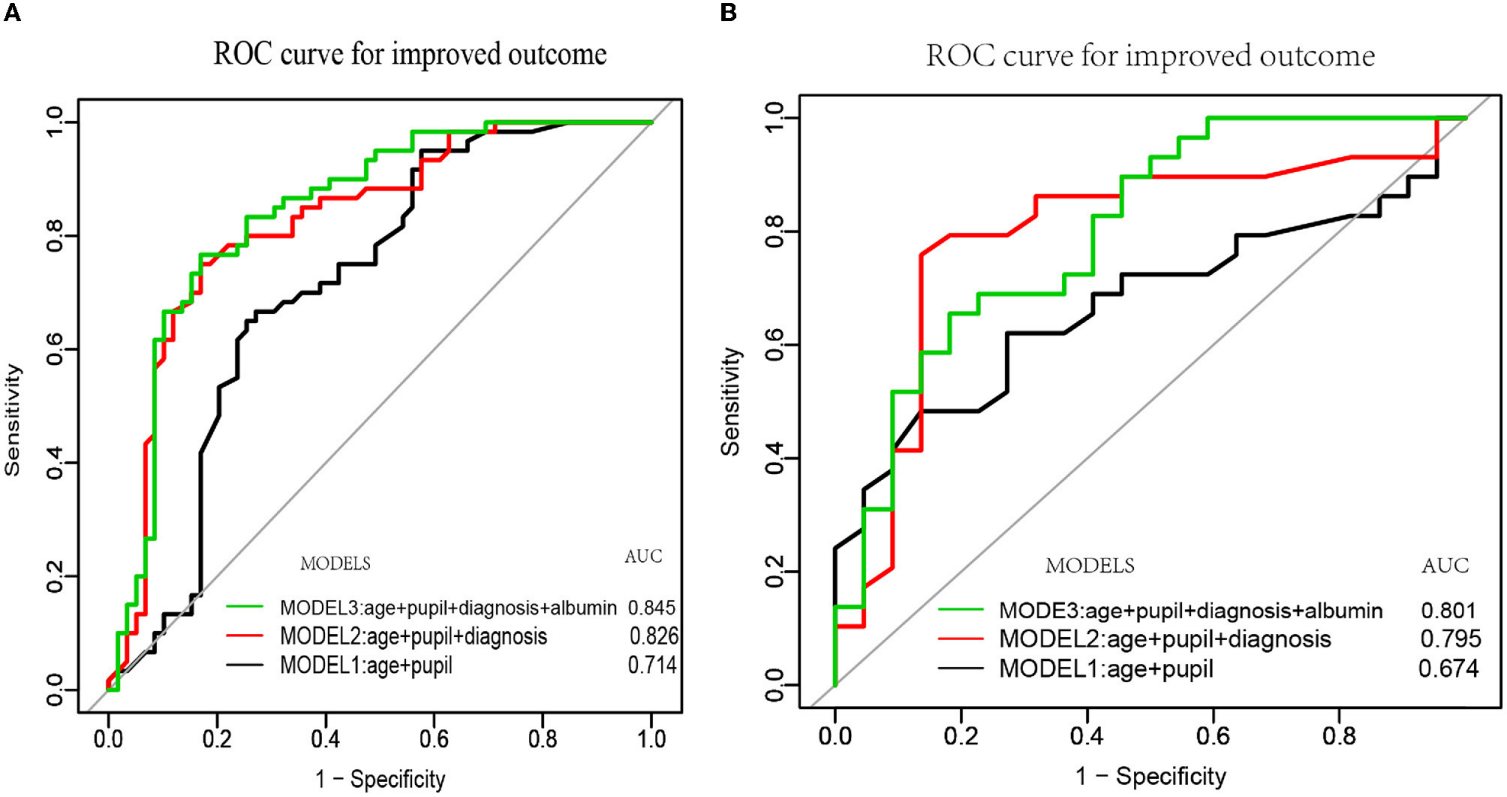
The ROC curves of age, pupil, diagnosis, albumin, and their combined models. MODEL 1 was constructed based on age and pupil reflex (black curve). MODEL 2 was established based on age, pupil reflex, and diagnosis (red curve). MODEL 3 was established based on age, pupil reflex, diagnosis, and serum albumin (green curve). **(A, B)** Depict the three ROC curves of the training and validation cohorts, respectively.

Previous studies reported the predictive value of age, pupil reflex, and diagnosis, but the value of serum albumin level has rarely been reported. We compared the performance of our model by incorporating age, pupil reflex, diagnosis, and serum albumin level with those of clinical index (CI) predictors (age+pupil) and CI predictors plus diagnosis in predicting improved clinical outcomes. The AUC of the receiver operating curve was evaluated for CI predictors (age + pupil), CI + entry diagnosis, and our final model (CI + entry diagnosis + serum albumin). Our results demonstrated that the AUC of MODEL3 (CI + entry diagnosis + serum albumin) was the highest in both the training and validation sets (AUC = 0.845 and 0.801, respectively) (Figure 5). We adjusted the model by sex, marital status, job, etiology, education level, hypertension, diabetes, and DOC duration.

The formula for the final model in the validation set was as follows: −0.97443 – 0.02752 ^*^ age + 0.94099 ^*^ (pupil reflex = presence) – 2.19467 ^*^ (diagnosis = VS) + 0.08060 ^*^ serum albumin in the training set. The formula for the final model in the validation set was as follows: −3.84513 – 0.01792 ^*^ age – 2.02802 ^*^ (diagnosis = VS) + 1.15271^*^(pupilreflex = presence) + 0.10959 ^*^ serumablumin.

## 4. Discussion

Prolonged disorders of consciousness, which is often caused by severe brain injury, always lead to severe complications (e.g., pneumonia, hemorrhage of the digestive tract, and dysfunction of the liver), poor clinical outcomes, and high mortality. Predicting clinical improvement in patients with pDOC is very helpful in supporting clinicians to offer suitable clinical care and aiding in the decision-making of patients and their family members (Scarpino et al., 2020). Although several previous studies aimed at identifying the risk factors that affect poor clinical outcomes, severe neurological deficit, or awakening from coma, only one nomogram model was established to predict the severe neurological deficit outcomes of pDOC (Kang et al., 2022). Thus, our present study aimed to build and validate a nomogram model for predicting improved clinical outcomes in patients with pDOC.

Many factors are known to influence the prognosis of patients with pDOC (Formisano et al., 2019). Our findings showed that younger age, MCS diagnosis at entry, higher serum albumin level, and the presence of pupillary reflex were associated with improved outcomes at 6 months after brain injury. Several studies showed that age, female sex (Estraneo et al., 2019), traumatic etiology (Whyte et al., 2009), and higher CRS-R (Portaccio et al., 2018; Foo et al., 2019) were related to better clinical outcomes. Whereas this predictive value of age was in line with our findings on patients with DOC, the value of female sex, traumatic etiology, and CRS-R were not. A study by Lucca et al. (2019) found that pDOC patients with CRS-R values >12 upon admission were associated with a favorable likelihood of emergence from DOC. In the current study, a majority of patients with CRS-R scores of <12 upon admission were presented, which may explain why CRS-R was not associated with improved outcomes in our study. Moreover, the results of the present study are consistent with those of a study (Estraneo et al., 2018), showing that a VS/UWS diagnosis was associated with a poor outcome and the total scores of CRS-R were not. In addition, a previous study by Lee et al. (2010) demonstrated that the presence of pupillary reflex had an effect on awakening from coma, which is consistent with our findings. They found that the AUC of pupil reflex in predicting severe neurological deficit in patients with coma was 0.744. One cause of DOC is damage to the ascending reticular activating system (ARAS) of the brainstem. The degree of brainstem damage is related to the level of consciousness, which has been known to clinicians. As pupillary light reflex is an important aspect of brainstem reflex, the bilateral absence of pupillary reflex indicates severe brainstem damage after injury. Therefore, we believe that pupil reflex is a good indicator to predict the outcomes in patients with pDOC.

Here, for the first time, we found that higher serum albumin level was associated with clinical improvement in patients with pDOC. A previous study (Chen et al., 2014) found that the concentration of serum albumin was associated with favorable outcomes in patients with traumatic brain injury. However, to the best of our knowledge, no research concerning the predictive value of serum albumin on clinical improvement in patients with pDOC has been reported until now. There are several reasons for the decrease in serum albumin levels in patients with DOC. First, the stress state leads to increased consumption of albumin. Second, intracranial hemorrhage and lung infection lead to albumin loss. In addition, the albumin from the blood passes into the cerebrospinal fluid (CSF) with the compromised blood–brain barrier (BBB) after severe brain injury (Kim et al., 2018). Finally, liver dysfunction in patients with pDOC results in decreased albumin synthesis. Therefore, a decrease in serum albumin implicates severe organ dysfunction, clinical instability, or severe brain injury, which may be the reason for poor clinical outcomes.

To the best of our knowledge, only one retrospective study established a nomogram model for predicting severe neurological deficit (including vegetate state and death) in patients with pDOC. Our present study would be the first prediction model for detecting the outcomes of patients with pDOC using a nomogram in a prospective study. The present prospective cohort study evaluated the prognostic value of clinical indices, entry diagnosis, and serologic markers that are easy to collect in patients with DOC 6 months after brain injury in grassroots hospitals. Therefore, we collected data on patients with pDOC within 1–3 months and established a simple, efficient, and accurate prediction model based on age, pupillary reflex, repeated diagnosis, and concentration of serum album. Considering the possible effect between clinical outcomes and the time of data collection, a covariate analysis of the time of data collection was performed. It showed that the time of data collection in our cohort study did not affect the clinical outcomes; OR 1.01 (95% CI 0.99–1.02; *p* = 0.48). Our cohort study demonstrated that the AUC of Model 3 (age + pupil + diagnosis + albumin) was the highest (AUC = 0.845) and was significantly higher than those of Model 1 (age + pupil) and Model 2 (age + pupil + diagnosis), the AUC values of which are 0.714 and 0.826, respectively. A retrospective prognostic model study (Kang et al., 2022) using age, diagnosis, GCS scores, and degree of brainstem auditory evoked potential (BAEP) demonstrated an AUC value of 0.815(29). Our final model (MODEL 3) showed an AUC value of 0.845. There are two reasons for the difference between the two models. First, GCS assessment is a good measure to evaluate the degree of brain damage of patients in the acute phase after severe brain injury rather than that of a patient in coma in the chronic phase (Bodien et al., 2021). Second, pupillary light reflex and BAEP examination are good indicators when evaluating brainstem injury, while the AUC value of pupil reflex (0.744) is good in predicting severe neurological deficits in patients with coma. Lee et al. (2010) stated that the AUC value was better in predicting awakening from coma (Fischer et al., 2004). Our study indicated that several feasible, cost-effective prognostic variables can provide an efficient and accurate prediction model for short-term clinical outcomes. However, the prognosis of pDOC is affected by many factors and remains a major clinical issue, especially as the prognostic values of the neuroimaging markers in predicting DOC are becoming a consensus among neurorehabilitation clinicians (Song et al., 2018; Yu et al., 2021). Thus, the values of other neurophysiologic or neuroimaging markers will be further investigated and integrated into the nomogram to improve the accuracy of the prediction model.

There are several limitations to our study. First, due to a small sample size, prognostic prediction was not allowed between the different diagnostic groups, which could underestimate the value of some predictors, such as CRS-R. Second, with the short follow-up time, identifying long-term prognostic markers was not sufficient as it has been found that prognostic markers may have different effects on each marker.

## 5. Conclusion

A higher concentration of serum albumin, higher consciousness level, young age, and presence of pupillary reflex could predict an improvement in pDOC 6 months after injury. These predictors are routinely measured in most hospitals, including grassroot rehabilitation units, and are not expensive and time-consuming. These feasible, cost-effective prognostic variables can provide an efficient and accurate prediction model for improved clinical outcomes and support clinicians by offering suitable clinical care and supporting the decision-making of patients and their family members.

## Data availability statement

The raw data supporting the conclusions of this article will be made available by the authors, without undue reservation.

## Ethics statement

The studies involving human participants were reviewed and approved by the First Affiliated Hospital of Nanchang University Ethics Committee. The patients/participants provided their written informed consent to participate in this study.

## Author contributions

QX conducted the experiment properly and prepared the manuscript. KL, YT, and XD are responsible for the management of patients. YZhon and YZhou performed repeated diagnoses and collected the data. YW performed the statistical analyses of the data. ZF secured funding for the project. All authors have read and approved the final manuscript.
